# Morphine Attenuates Apically-Directed Cytokine Secretion from Intestinal Epithelial Cells in Response to Enteric Pathogens

**DOI:** 10.3390/pathogens3020249

**Published:** 2014-04-02

**Authors:** Amanda J. Brosnahan, Bryan J. Jones, Cheryl M. Dvorak, David R. Brown

**Affiliations:** 1 Department of Veterinary and Biomedical Sciences, College of Veterinary Medicine, University of Minnesota, 1988 Fitch Avenue, Saint Paul, MN 55108-6010, USA; E-Mails: jone0803@umn.edu (B.J.J.); dvora013@umn.edu (C.M.D.); 2 Department of Science, Concordia University, 1282 Concordia Avenue, Saint Paul, MN 55104-5494, USA; E-Mail: brosnahan@csp.edu

**Keywords:** morphine, opioid receptors, small intestine, epithelial cell, *Escherichia coli*, *Salmonella enterica*, interleukin-6, interleukin-8, Toll-like receptors, swine

## Abstract

Epithelial cells represent the first line of host immune defense at mucosal surfaces. Although opioids appear to increase host susceptibility to infection, no studies have examined opioid effects on epithelial immune functions. We tested the hypothesis that morphine alters vectorial cytokine secretion from intestinal epithelial cell (IPEC-J2) monolayers in response to enteropathogens. Both entero-adherent *Escherichia coli* O157:H7 and entero-invasive *Salmonella enterica* serovar Typhimurium increased apically-directed IL-6 secretion and bi-directional IL-8 secretion from epithelial monolayers, but only IL-6 secretion evoked by *E. coli* was reduced by morphine acting through a naloxone-sensitive mechanism. Moreover, the respective type 4 and 5 *Toll*-like receptor agonists, lipopolysaccharide and flagellin, increased IL-8 secretion from monolayers, which was also attenuated by morphine pretreatment. These results suggest that morphine decreases cytokine secretion and potentially phagocyte migration and activation directed towards the mucosal surface; actions that could increase host susceptibility to some enteric infections.

## 1. Introduction

Morphine and other opioid receptor agonists alter systemic innate and adaptive immune responses and increase host susceptibility to infection [1]. For example, impairments in neutrophil function, macrophage phagocytosis, T lymphocyte cytokine production, and B cell antigen presentation occur after exposure to morphine. In some cases, these effects are mediated through mu-, delta- or kappa-opioid receptors (MOR, DOR, and KOR, respectively), transcripts of which have been detected in human immune cell lines and primary cells [1,2]. Morphine treatment has been associated with increased translocation of bacterial commensals or their associated molecular patterns (e.g., lipopolysaccharide) from the gut into the systemic lymphatic and blood circulation [3,4,5,6,7]. Moreover, animals treated with morphine exhibit greater susceptibility to enteric infections, including those produced by *Salmonella enterica* serovars Enteritidis and Typhimurium, *Listeria monocytogenes*, *Vibrio cholerae*, and *Pseudomonas aeruginosa* [8,9,10,11].

Epithelial cells constitute the first line of innate host defense in mucosal tissues and possess accessory immune functions [12]. In the gastrointestinal tract, epithelial cells in the intestinal mucosa appear to be innervated by opioid peptide-immunoreactive submucosal nerves and can interact with food-derived opioids such as casomorphins that are generated in the bowel lumen [13,14,15]. There is evidence that intestinal epithelial cells from several families of animals may express specific opioid binding sites [16,17,18,19,20,21,22]. The effects of morphine or other opioids on epithelial immune responses to pathobionts or pathogenic microorganisms have not been hitherto investigated. IPEC-J2 cells derived from the neonatal swine small intestine have been used extensively to study host-pathogen interactions between epithelial cells and entero-invasive or entero-adherent bacterial pathogens [23].

In this study, we utilized confluent, polarized IPEC-J2 cell monolayers to test the hypothesis that morphine modifies vectorial epithelial immune responses to entero-invasive *S.* Typhimurium or entero-adherent *E. coli* O157:H7, and their associated molecular patterns, *i.e.* flagellin and lipopolysaccharide.

## 2. Results and Discussion

### 2.1. Effect of Morphine on Epithelial Immune Responses to Enteric Pathogens

**Figure 1:**
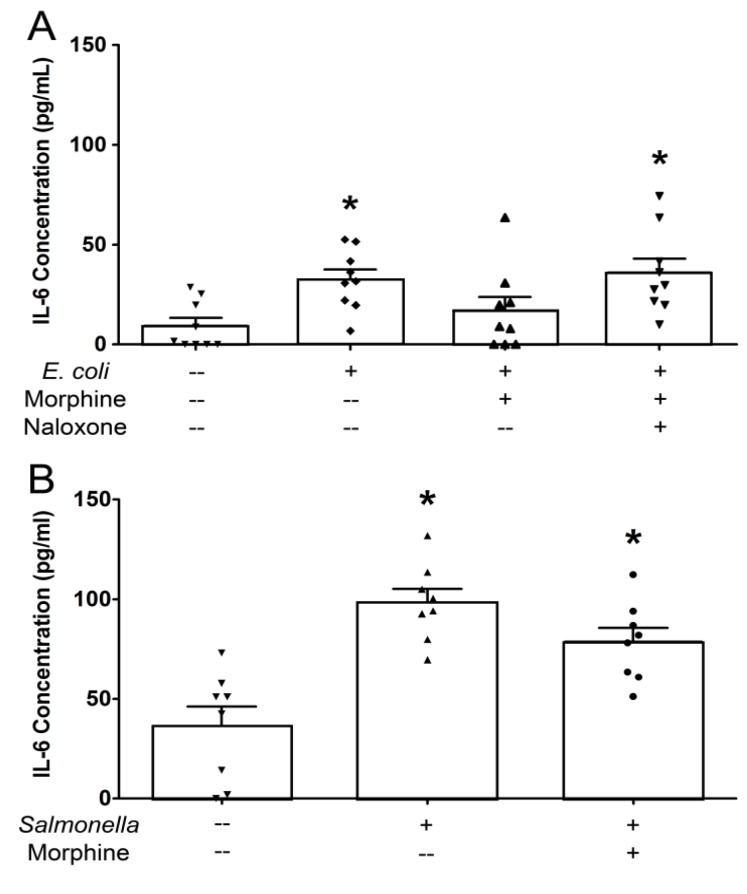
Morphine attenuates apical IL-6 secretion in IPEC-J2 monolayers exposed to entero-adherent *E. coli* or entero-invasive salmonellae. IPEC-J2 cell monolayers were grown to confluency in Transwells. Morphine (10 μM) ± naloxone (1 μM) was added to the basolateral medium 30 minutes before the addition of *E. coli* O157:H7 or *S.* Typhimurium (10^7^ colony-forming units (CFUs)) to the apical medium. After a 6 hr incubation, the apical medium was collected and analyzed by ELISA for IL-6. (A) *E. coli.* *Significantly higher than apical medium-only control by one-way ANOVA [*F*(3,32) = 4.716, *p* = 0.0078] and Tukey's *post-hoc* test. N = 9 replicates (one condition/monolayer in each of nine different experiments). (B) *S.* Typhimurium. *Significantly higher than apical medium-only control by one-way ANOVA [*F*(2,21) = 15.78, *p* < 0.0001] and Tukey’s *post-hoc* test. N = 8 replicates (one condition/monolayer in each of eight different experiments).

Interleukin-6 secretion was significantly increased in the apical media bathing epithelial monolayers exposed to *E. coli* or *S.* Typhimurium for 6 hr relative to monolayers unexposed to the bacteria (Figure 1). In addition, these two pathogenic bacteria evoked increases in bi-directional IL-8 secretion from the monolayers (Table 1). The present results with *S*. Typhimurium are in concordance with those reported previously by Skjolaas *et al.* [24,25], IPEC-J2 cell monolayers released the cytokine IL-6 into the apical (mucosal) medium and the chemokine IL-8 into both the apical and basolateral (serosal) media after bacterial exposure. On the other hand, the finding that strain 85-170 of Shiga toxin-negative *E. coli* O157:H7 elicits vectorial secretion of IL-6 and IL-8 from IPEC-J2 monolayers has not been hitherto reported.

Morphine pretreatment significantly decreased IL-6 secretion from *E. coli*-treated monolayers by a naloxone-sensitive mechanism (Figure 1A), but did not decrease IL-6 secretion in response to *S.* Typhimurium (Figure 1B). The apparent lack of morphine effect on the relatively large cytokine responses of monolayers to *S*. Typhimurium, an entero-invasive pathogen, suggests that either *E. coli* and *Salmonella* may be acting through different intracellular signaling pathways to elicit directional cytokine secretion or that the more robust effects of *Salmonella* on epithelial interleukin secretion could not be attenuated by morphine, at least at the concentration tested. In a separate group of six experiments, the anti-inflammatory steroid dexamethasone significantly blunted the increase in apical IL-6 secretion evoked by *Salmonella* exposure (118.35 ± 9.60 pg/mL IL-6 *versus* 69.14 ± 8.12 pg/mL IL-6 in monolayers untreated or pre-treated with dexamethasone respectively, N = 6 replicates, analyzed by Student’s *t* test, *p* < 0.01). Dexamethasone had no effect on IL-8 secretion (data not shown). In human corneal and bronchial epithelial cells, dexamethasone reportedly inhibits both IL-6 and IL-8 secretion evoked by pro-inflammatory mediators [26,27,28]. Morphine alone or in combination with naloxone did not modify constitutive cytokine release from cell monolayers that were unexposed to bacteria and did not alter epithelial IL-8 responses to either pathogen (data not shown).

**Table 1:** Effects of *E. coli* 0157:H7 and *S*. *enterica* Typhimurium on Polarized IL-8 Secretion from IPEC J2 Cell Monolayers.

	Control	*E. coli*	*p* value	Control	*S.* Typhimurium	*p* value
Apical IL-8 concentration	101.92 ± 4.54	425.60 ± 20.12	0.0015	143.72 ± 16.98	1129.74 ± 50.37	<0.001
Basolateral IL-8 concentration	16.68 ± 2.78	1188.47 ± 45.02	<0.001	48.14 ± 14.43	2516.64 ± 66.17	<0.001

Values are expressed as mean ± S.E. of IL-8 concentration in pg/mL of apical or basolateral bathing medium (N = one condition/monolayer in each of 9 and 8 experiments with *E. coli* and *S*. Typhimurium, respectively). P values were determined by Student’s *t* test comparing IL-8 concentrations in apical or basolateral media bathing monolayers that were unexposed (control) or exposed to bacteria.

### 2.2. Effects of Morphine on Immune Responses by Epithelial Monolayers Exposed to Pathogen-Associated Molecular Patterns

**Figure 2:**
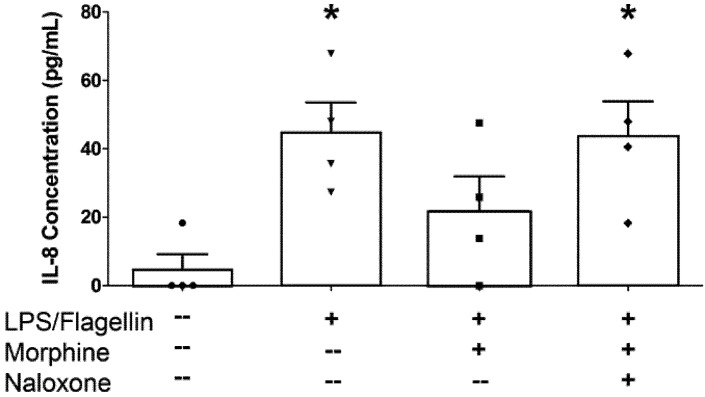
Morphine attenuates apical IL-8 secretion in IPEC-J2 monolayers exposed to lipopolysaccharide (LPS) and flagellin. IPEC-J2 cell monolayers were grown to confluency in Transwells. Morphine (10 μM) ± naloxone (1 μM) was added to the basolateral medium 30 minutes before the addition of LPS (1 μg/mL) to the apical medium and flagellin (100 ng/mL) to the basolateral medium. After a 6 hr incubation, the apical medium was collected and analyzed by ELISA for IL-8. * Significantly different than medium-only control by one-way ANOVA [*F*(3,12) = 4.853, *p* = 0.0195] and Tukey’s *post-hoc* test. N = 4 replicates (one condition/monolayer in each of four different experiments).

The bacterial products lipopolysaccharide (LPS) and flagellin, agonists at TLR-4 and TLR-5 respectively, stimulated bi-directional IL-8, but not IL-6 secretion from epithelial monolayers when administered in combination (Figure 2). Their combined effect on apical IL-8 secretion was some 10 to 30 fold less than that evoked by exposure of monolayers to *E. coli* or *S.* Typhimurium. Apically-directed IL-8 secretion in response to the TLR agonists was inhibited by morphine, an effect that was sensitive to naloxone (Figure 2).

Morphine pretreatment had no effect on TLR-elicited basolateral IL-8 secretion (data not shown). The chemokine IL-8 plays an important role in recruiting neutrophils to the intestinal epithelial surface [29]. Morphine and other opioid agonists have been reported to interact non-competitively with TLR-4 in glia and other immuno-competent cells at a naloxone-insensitive site of action [30,31]. In the present study, the naloxone-sensitive effect of morphine in decreasing LPS-induced IL-8 secretion is likely due to an opioid receptor-dependent mechanism and not to direct interactions with TLR-4. If morphine actions are extrapolated to the extensive surface area of the intestinal mucosa, we hypothesize that reductions in IL-8 or IL-6 concentrations at the mucosal surface might impair neutrophil chemotaxis and activation, leading to an increased susceptibility to mucosal infection with potential bacterial translocation and sepsis [32,33].

## 3. Experimental Section

### 3.1. Reagents

Porcine jejunal epithelial IPEC-J2 cells were a gift from Bruce Schultz (Department of Anatomy and Physiology, College of Veterinary Medicine, Kansas State University, Manhattan, KS, USA). Cells were cultured as previously described [23].

A swine isolate of *Salmonella enterica* serovar Typhimurium DT104 was provided by Jeffrey Bender (Center for Animal Health and Food Safety, University of Minnesota, St. Paul, MN, USA) and has been previously described [34]. Strain 85–170 of Shiga toxin-negative *Escherichia coli* O157:H7 was provided by Mark Stevens (Roslin Institute and Royal (Dick) School of Veterinary Studies, University of Edinburgh, Midlothian, EH25 9RG Scotland, UK) and has also been previously described [35,36]. All bacteria were grown in Luria-Bertani (LB) broth at 37 °C with 5% CO_2_.

Morphine sulfate was obtained from the National Institute on Drug Abuse (Bethesda, MD, USA). Naloxone, dexamethasone and lipopolysaccharide (LPS, purified from *S.* Typhimurium) were obtained from Sigma-Aldrich (St. Louis, MO, USA). Flagellin was purchased from Enzo Life Sciences (Farmingdale, NY, USA).

### 3.2. IPEC-J2 Experiments

Cells were grown to confluency on 0.4 μm pore-size Transwell^®^ filters (Corning, Tewksbury, MA) and tested for trans-epithelial electrical resistances (TEERs) >700 Ω, indicative of a confluent monolayer. Cell monolayers were washed and incubated in antibiotic-free medium one day prior to experimentation. On the day of experimentation, TEERs of >400 Ω across each monolayer were confirmed. Morphine (10 μM) ± naloxone (1 μM) was added to the basolateral medium for 30 minutes prior to the addition of bacteria (10^7^ colony-forming units, CFUs) to the apical medium. In one set of experiments, LPS (1 μg/mL) was added to the apical medium instead of bacteria with the simultaneous addition of flagellin (100 μg/mL) to the basolateral medium. These concentrations of LPS and flagellin chosen were previously reported to evoke maximal chemokine release from cultured IPEC-J2 cells [37] and human HT29 colonic adenocarcinoma cells [38], respectively. Some experiments with dexamethasone (1 μM), an anti-inflammatory glucocorticoid, were also conducted; it was added to the basolateral medium for 30 minutes prior to the addition of bacteria (10^7^ CFUs) to the apical medium. All experiments were carried out over 6 h, at which point apical and basolateral media were collected, centrifuged to remove bacteria, and frozen at −20 °C until ELISA analysis. ELISA analysis of secreted proteins was determined using porcine Quantikine kits (R&D Systems, Minneapolis, MN).

## 4. Conclusions

In the present study, morphine decreased apical secretion of IL-6 or IL-8 from porcine intestinal epithelial cells in response to entero-adherent *E. coli* O157:H7 or bacteria-associated molecular patterns, respectively. To our knowledge, this is the first report of an opioid action on immune responses in non-transformed intestinal epithelial cells *in vitro*. However, investigations *in vivo* indicate that MOR agonists decrease injury and inflammatory responses in intestinal epithelial cells [39,40,41]. The effects of morphine were inhibited by the general opioid receptor antagonist naloxone, suggesting that they are mediated by opioid receptors. Indeed, mRNAs for MOR and DOR have been detected at low levels of expression in IPEC-J2 cells (A.J. Brosnahan and D.R. Brown, unpublished results). The opioid alkaloid preferentially interacts with MOR, but can interact with DOR at higher concentrations [42]. Although the opioid receptor type(s) and mechanisms underlying the action of morphine were not investigated here, inhibitory interactions between opioid agonists and pro-inflammatory transcription factors, notably nuclear factor kappaB, have been reported to occur in leukocytes [43,44,45,46]. Future studies should be designed to explore this and other possible mechanisms of opioid action in epithelial cells.

Epithelial cells comprise the first line of defense against potential pathogens in the gut. They constitute a physical barrier against microbial penetration and detect invading microorganisms and communicate to neighboring innate immune cells through the production and vectorial release of various cytokines and chemokines. Perturbations in the process of epithelial cell to immunocyte communication by opiates such as morphine and perhaps other drugs used in the palliative care of immuno-compromised patients may further impair disease resistance and worsen the subsequent course of intestinal infections.
